# Development of a High-Throughput Analytical Method for Antimicrobials in Wastewater Using an Automated Pipetting and Solid-Phase Extraction System

**DOI:** 10.3390/antibiotics13040335

**Published:** 2024-04-05

**Authors:** Takashi Azuma, Nobuaki Matsunaga, Norio Ohmagari, Makoto Kuroda

**Affiliations:** 1 Department of Pharmacy, Osaka Medical and Pharmaceutical University, Takatsuki 569-1094, Japan; 2 AMR Clinical Reference Center, National Center for Global Health and Medicine, Tokyo 162-8655, Japan; nomatsunaga@hosp.ncgm.go.jp (N.M.); nohmagari@hosp.ncgm.go.jp (N.O.); 3 Disease Control and Prevention Center, National Center for Global Health and Medicine, Tokyo 162-8655, Japan; 4 Pathogen Genomics Center, National Institute of Infectious Diseases, Tokyo 162-8640, Japan

**Keywords:** antimicrobials, hospital wastewater, high-throughput analysis (HTA), automated pipetting and solid-phase extraction (SPE), antimicrobial resistance (AMR)

## Abstract

Antimicrobial resistance (AMR) has emerged and spread globally. Recent studies have also reported the presence of antimicrobials in a wide variety of aquatic environments. Conducting a nationwide monitoring survey of AMR in the environment to elucidate its status and to assess its impact on ecosystems and human health is of social importance. In this study, we developed a novel high-throughput analysis (HTA) system based on a 96-well plate solid-phase extraction (SPE), using automated pipetting and an SPE pre-treatment system. The effectiveness of the system as an HTA for antimicrobials in environmental water was verified by comparing it with a conventional manual analytical system in a domestic hospital over a period of two years and four months. The results of the manual analysis and HTA using a combination of automated pipetting and SPE systems were generally consistent, and no statistically significant difference was observed (*p* > 0.05) between the two systems. The agreement ratios between the measured concentrations based on the conventional and HTA methods were positively correlated with a correlation coefficient of *r* = 0.99. These results indicate that HTA, which combines automated pipetting and an SPE pre-treatment system for rapid, high-volume analysis, can be used as an effective approach for understanding the environmental contamination of antimicrobials at multiple sites. To the best of our knowledge, this is the first report to present the accuracy and agreement between concentrations based on a manual analysis and those measured using HTA in hospital wastewater. These findings contribute to a comprehensive understanding of antimicrobials in aquatic environments and assess the ecological and human health risks associated with antimicrobials and antimicrobial-resistant bacteria to maintain the safety of aquatic environments.

## 1. Introduction

The emergence and spread of antimicrobial-resistant bacteria (AMRB) is progressing globally [1,2,3]. Antimicrobial resistance (AMR) is not limited to the medical field but is closely linked to animal husbandry, agriculture, fisheries, and other aspects of our daily lives [4,5]. Aquatic environments are interconnected between cities and nature. Water resources, including drinking water and urban rivers that receive treated wastewater generated by human activities, play an important role in human habitation and public health [6,7,8].

Recent studies have shown that AMRB and antimicrobial-resistant genes (AMRGs), which pose a threat to human and animal health, are found in wastewater from wastewater treatment plants (WWTPs), hospitals, and other medical facilities as well as in wastewater from livestock operations [9,10]. These sources have been attributed to AMR, which is prevalent in humans and animals [11,12]. Additionally, AMRB and AMRGs are found in aquatic environments, such as rivers, lakes, and oceans, and research is underway to assess their environmental and human health risks [13,14,15]. Various pharmaceutical ingredients also remain in wastewater, and antimicrobials have been detected. This is mainly due to the excretion of antimicrobials from the body after administration. These pollutants have been detected in aquatic environments such as rivers, lakes, and marine areas [16,17]. Antimicrobials have toxic effects on aquatic ecosystems, posing a potential risk for the emergence and spread of new AMRB by the microorganisms present in the environments [18,19,20]. Monitoring of antimicrobials in wastewater can be useful for understanding the status of AMR in the environment. Establishing a system for conducting nationwide monitoring surveys will provide useful information for clarifying the impact of AMR on the environments [21,22,23]. However, surveillance methods for AMR and antimicrobials in the environment are still in the early stages of global development, and many unknown factors remain in Japan [24,25,26,27]. Therefore, an urgent need prevails to establish a survey method to quantify and estimate the environmental risk to humans and animals and elucidate AMR mechanisms and transmission pathways [28,29].

To date, a highly sensitive simultaneous multiresidue analytical method, combining solid-phase extraction (SPE) and liquid chromatography–tandem mass spectrometry (LC–MS/MS), has been widely used to measure antimicrobial concentrations in environmental waters [30,31,32]. This measurement method offers high accuracy and sensitivity and is highly versatile. In contrast, SPE, a sample pre-treatment operation, is generally performed manually by humans, and the SPE instruments and related products available on the market are mainly syringe barrel-type products for measuring one sample at a time or cartridge-type products for connecting syringes [33,34]. Therefore, to establish a system for the nationwide development of environmental water monitoring surveys for AMR, developing and implementing a high-throughput analysis (HTA) system that can process multiple samples simultaneously and that excels in speed and accuracy is essential [35,36]. Research and development in the field of microbiology have progressed rapidly, introducing robotic manipulation as a replacement for multi-sample processing, including in clinical laboratories [37,38,39]. Previous studies have reported on reporter gene assays for endocrine disruptors [40] and the detection of AMRGs [41,42] in environmental waters. However, few studies have analysed chemical substances in this regard (i.e., the measurement of antimicrobials in the environment) because chemical measurement systems are complex and require numerous laboratory instruments. The number of cases studied in each country is limited to a few regions, and a national overview has not yet been compiled [43,44]. Therefore, the successful development of an HTA system with excellent speed and accuracy will make it possible to conduct nationwide monitoring of antimicrobials in environmental waters for comprehensively elucidating the current status of AMR, which has been difficult in the past [23,45,46].

The remarkable development of science and technology in recent years has led to the development of automated pipetting systems and robotic technology with higher performance [47,48]. Given these advancements, the first devices to automate pre-treatment equipment using SPE are expected to be sold in the second half of 2022 [49]. Moreover, the application of SPE has expanded beyond the environmental field to include the determination of blood concentrations of pharmaceuticals and drug discovery assays. Currently, 96-well plate SPE devices that can process multiple samples simultaneously are now available on the market, in addition to conventional SPE devices that process one sample at a time. These instruments are now commercially available, and studies have been conducted using them [48,50,51]. Therefore, we developed an HTA based on a 96-well plate SPE that fully utilised the combination of an automated pipetting system and an automated SPE system to create a novel monitoring system for antimicrobials in the environmental waters. In this study, the effectiveness of the developed HTA system for antimicrobials in environmental water was verified through a comparative evaluation. Then, the evaluation compared the conventional manual analytical system and the developed HTA system, which was adapted for a monitoring study using actual hospital wastewater, to assess the effectiveness of the developed methods for the evaluation of the occurrence and environmental risk of antimicrobials in water environments.

## 2. Materials and Methods

### 2.1. Chemicals and Reagents

A total of 17 antimicrobials were evaluated in five categories, as shown in Table 1. The categories that were analysed were *β*-lactams (ampicillin, benzylpenicillin, cefdinir, cefpodoxime, cefpodoxime proxetil, and ceftiofur), new quinolones (ciprofloxacin, enrofloxacin, and levofloxacin), macrolides (azithromycin and clarithromycin), tetracyclines (chlortetracycline, doxycycline, minocycline, oxytetracycline, and tetracycline), and glycopeptide (vancomycin). This selection was based on previous reports of their occurrence and frequency of detection in hospital wastewater, WWTPs, and river water both in Japan and worldwide [52,53,54], and antimicrobial use in medical settings in Japan [55,56]. All analytical standards were of high purity (>98% purity) and were purchased from Cayman Chemical (Ann Arbor, MI, USA), LKT Laboratories (St. Paul, MN, USA), Santa Cruz Biotechnology, Inc. (Santa Cruz, CA, USA), Tokyo Chemical Industry Co., Ltd. (Tokyo, Japan), and Toronto Research Chemicals, Inc. (Toronto, ON, Canada). Individual standard stock solutions (10 mg/L) were prepared in methanol depending on the purity of each reagent and stored at −20 °C. All aqueous solutions were prepared with ultrapure water (18.2 MΩ·cm) provided by a Milli-Q purification system (MilliporeSigma, Watford, UK). LC–MS-grade methanol, acetone, formic acid, reagent-grade ascorbic acid, and hydrochloric acid were purchased from the FUJIFILM Wako Pure Chemical Corporation (Osaka, Japan).

### 2.2. Analytical Procedures for Antimicrobials in the Environmental Water Based on the Manual Analysis

The manual analysis of target antimicrobials in wastewater was conducted using a combination of SPE and ultra-performance liquid chromatography–tandem mass spectrometry (UPLC–MS/MS), as previously described [57,58]. Briefly, 10 mL of wastewater was filtered through a glass fibre filter (GF/B, pore size of 1 μm, Whatman, Maidstone, UK). The filtered solutions were then passed through OASIS HLB syringe barrel cartridges with a 200 mg solid-phase carrier and a 6 mL column (Waters Corp., Milford, MA, USA), which had been preconditioned with 3 mL of methanol and 3 mL of Milli-Q water adjusted to pH 3 with 1 N hydrochloric acid at a flow rate of 1 mL/min. All the cartridges were cleaned by washing with 6 mL of Milli-Q water, then pre-adjusted to pH 3 and dried using a vacuum pump. Finally, the adsorbed antimicrobials were eluted with 6 mL of a 1:1 (*v*/*v*) mixture of acetone and methanol and then mildly evaporated to dryness under a gentle stream of N_2_ gas at 37 °C. The residue was solubilised in 200 μL of a 90:10 (*v*/*v*) mixture of 0.1% formic acid in methanol. Subsequently, 10 μL of this solution was analysed using a UPLC–MS/MS fitted with a column (2.1 mm × 100 mm, 1.7 μm) UPLC BEH C_18_ (Waters Corp.) coupled to a tandem quadrupole mass spectrometer (Waters Corp.).

**Table 1:** Chemical structures and physicochemical properties of the target antimicrobials examined [59].

Classification	CAS Registry Number	Antimicrobials	Molecular Formula	Molecular Mass (g/mol)	Structure	p*K*_a_	Log*P*
*β*-lactams	69-53-4	Ampicillin (APL)	C_16_H_19_N_3_O_4_S	349.4	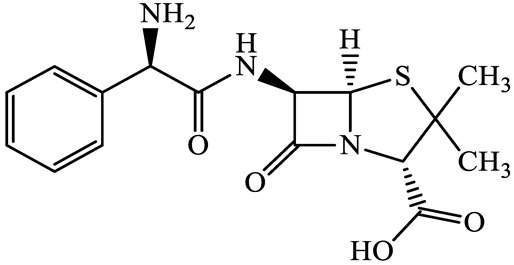	2.4	1.4
	61-33-6	Benzylpenicillin (BZP)	C_16_H_18_N_2_O_4_S	334.4	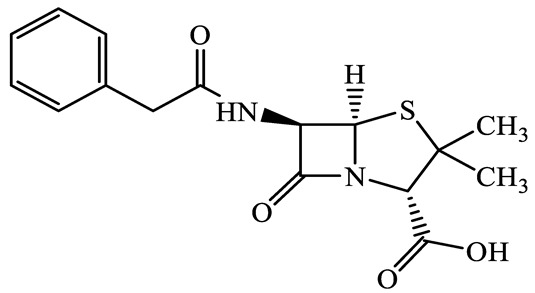	2.5	1.7
	91832-40-5	Cefdinir (CDN)	C_14_H_13_N_5_O_5_S_2_	395.4	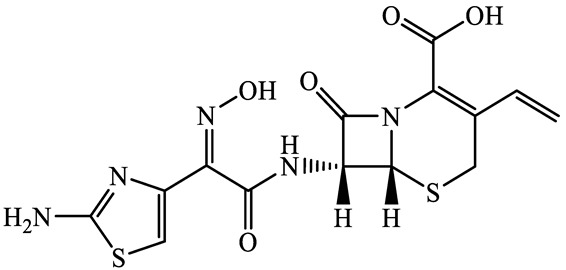	2.8	−1.8
	80210-62-4	Cefpodoxime (CPX)	C_17_H_19_N_5_O_6_S_2_	453.5	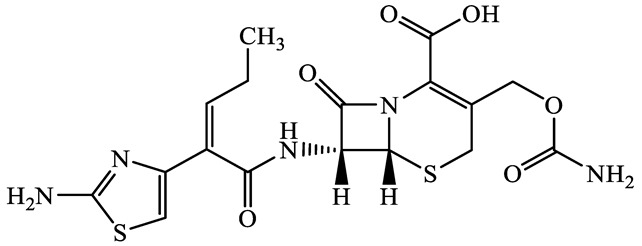	2.8 (Acid) 1.7 (Base)	0.4
	87239-81-4	Cefpodoxime proxetil (CPXP)	C_21_H_27_N_5_O_9_S_2_	557.6	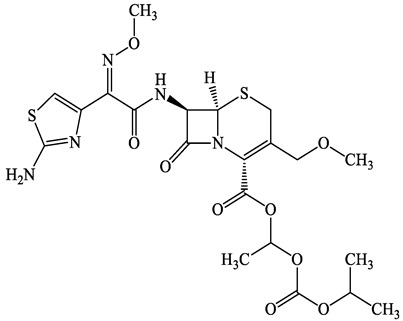	8.1 (Acid) 1.7 (Base)	2.9
	80370-57-6	Ceftiofur (CTF)	C_19_H_17_N_5_O_7_S_3_	523.6	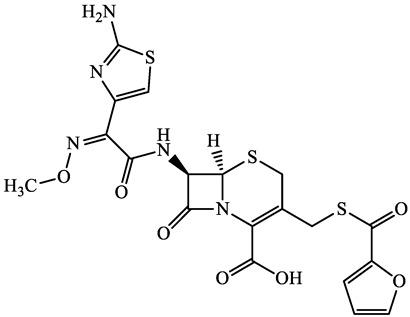	2.6	1.7
New quinolones	85721-33-1	Ciprofloxacin (CFX)	C_17_H_18_FN_3_O_3_	331.3	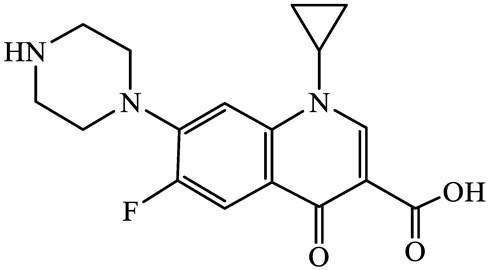	6.0	1.3
	93106-60-6	Enrofloxacin (EFX)	C_19_H_22_FN_3_O_3_	359.4	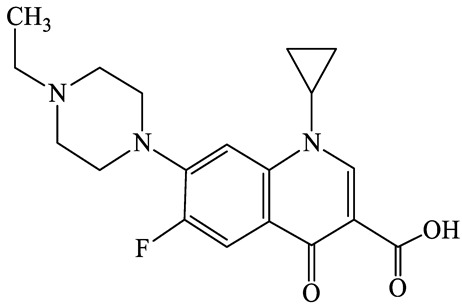	6.4	2.3
	100986-85-4	Levofloxacin (LFX)	C_18_H_20_FN_3_O_4_	361.4	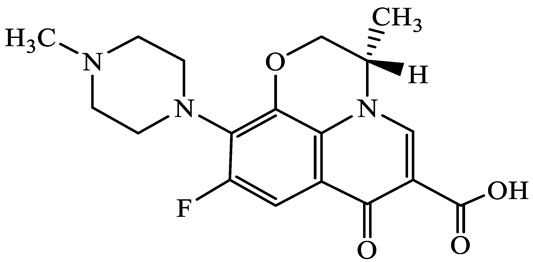	5.2	1.6
Macrolides	83905-01-5	Azithromycin (ATM)	C_38_H_72_N_2_O_12_	749.0	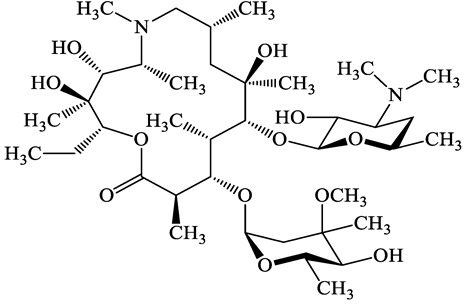	13.3	3.3
	81103-11-9	Clarithromycin (CTM)	C_38_H_69_NO_13_	748.0	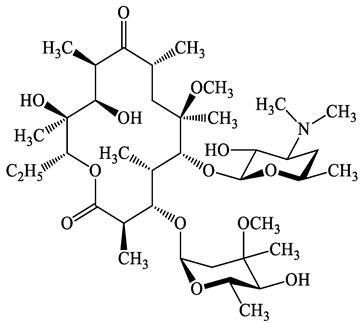	13.1	3.2
Tetracyclines	57-62-5	Chlortetracycline (CTCL)	C_22_H_23_ClN_2_O_8_	478.9	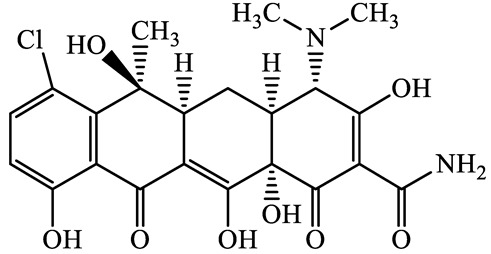	4.5	4.8
	564-25-0	Doxycycline (DCL)	C_22_H_24_N_2_O_8_	444.4	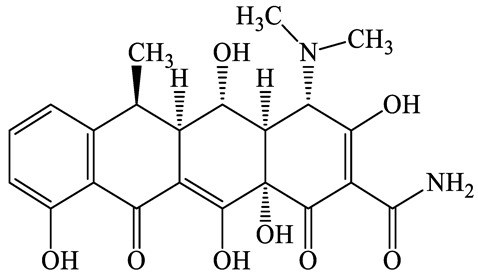	4.5 (Acid) 10.8 (Base)	1.8
	10118-90-8	Minocycline (MCL)	C_23_H_27_N_3_O_7_	457.5	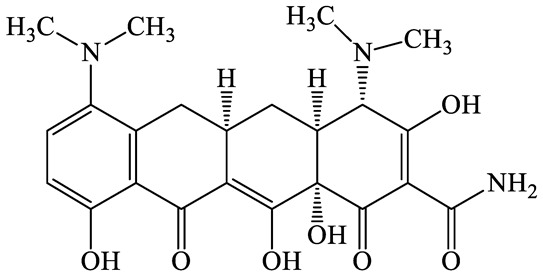	4.5 (Acid) 11.1 (Base)	2.2
	79-57-2	Oxytetracycline (OTCL)	C_22_H_24_N_2_O_9_	460.4	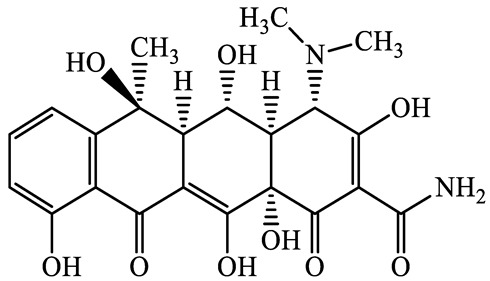	4.5	−1.5
	60-54-8	Tetracycline (TCL)	C_22_H_24_N_2_O_8_	444.4	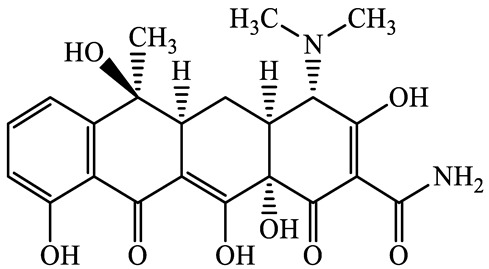	4.5	−1.5
Glycopeptide	1404-90-6	Vancomycin (VMC)	C_66_H_75_Cl_2_N_9_O_24_	1449.3	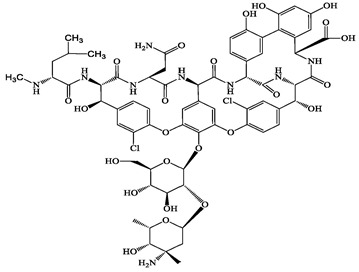	3.0	−1.4

CAS: chemical abstracts service; p*K*_a_: acid dissociation constant; Log*P*: octanol-water partition coefficients.

### 2.3. Development of Analytical Procedures for Antimicrobials in Environmental Water Based on the HTA Systems Using Automated Pipetting Equipped with Automated SPE

**Figure 1:**
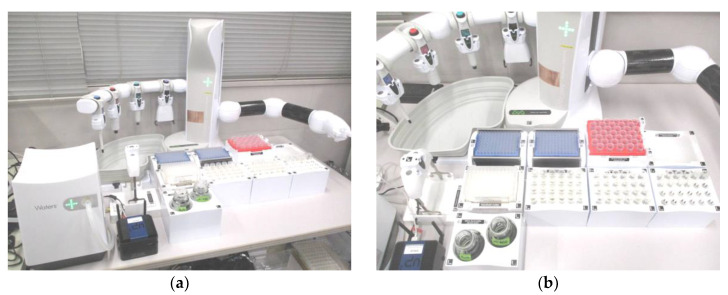
The HTA system, combining an automated aliquoter and an automated SPE system, used in this study: (**a**) overview of the HTA system and (**b**) enlarged detail of each HTA component.

An HTA of the target antimicrobials in the wastewater was performed using 96-well SPE plates, automated pipetting, and automated SPE systems based on manual analysis. Specifically, wastewater samples filtered through the glass fibre filter described in Section 2.2 were passed through an OASIS HLB 96-well plate (Waters Corp.). Various solid-phase volumes of 96-well SPE plates (5, 10, 30, and 60 mg) are currently available on the market [60]. In this study, larger volumes of the solid phase (30 and 60 mg) were investigated to match the solid-phase volume used in manual measurements (200 mg). Automated pipetting during the SPE procedure was performed using the Andrew + Pipetting Robot (Waters Corp.), whereas vacuum pumping and dehydration of the liquid to solid phase were performed using the Extraction+ Base Kit WITH Gripper (Waters Corp.) [49]. The appearance and configuration of the HTA using automated pipetting equipped with the automated SPE systems used in this study is shown in Figure 1. The adsorbed antimicrobials were eluted with a 1:1 (*v*/*v*) mixture of acetone and methanol and then mildly evaporated to dryness under a gentle stream of N_2_ gas at 37 °C. The residue was solubilised in 100 μL of a 90:10 (*v*/*v*) mixture of 0.1% formic acid in methanol. Finally, 10 μL of this solution was subjected to UPLC–MS/MS analysis, as described in Section 2.2.

In the present investigation, to optimise the SPE process using a 96-well SPE plate, the effects of various measurement conditions on the amount of sample water loading to solid-phase (2.5 mL, 5 mL, and 10 mL), elution volume (50 μL, 100 μL, 500 μL, 1000 μL), filtration, and on the peak intensity of each antibacterial agent in the solvent composition of the final solvent composition for LC–MS/MS measurements were investigated using untreated wastewater influent of a WWTP located in urban areas.

### 2.4. Method Validation

Six-point standard calibration curves were constructed for quantification ranging from 0.5 to 200 ng/mL in a 90:10 (*v*/*v*) mixture of 0.1% formic acid in methanol. Individual linear calibration curves for each compound were obtained in the concentration range 0.5 of 200 ng/mL (*r*^2^ > 0.99), using a weighting factor of 1/x. Instrument control and quantification were performed using the Mass Lynx 4.1 software (Waters Corp.).

Quantification involved subtracting the blank data from the corresponding data obtained from the spiked sample solutions to account for matrix effects and losses during sample extraction [61,62]. Similarly, the recovery rates were calculated from the deviations between the spiked and standard calibration data [63,64]. The results of various measurement conditions using a syringe-barrel SPE cartridge and a 96-well SPE plate were evaluated based on the distribution of the recovery rates. The degree of recovery rate was compared to the increment percentage by grouping the data into four types: ≥70% (favourable), >40% to ≥70% (measurement possible), >40% to ≥20% (near measurable limit), and <20% (measurement not possible), based on previous reports on the analysis of pharmaceuticals and other emerging pollutants in environmental waters [62,65,66]. The limits of detection and quantification for the water samples were calculated based on the concentrations at signal-to-noise ratios of 3 and 10, respectively, according to methods applied to pharmaceuticals in river water and wastewater samples [67,68]. The validation of the detection and quantification methods used for the UPLC–MS/MS analysis and the limits of detection (LODs) and limits of quantification (LOQs), and reproducibility (%) (*n* = 3) are shown in Table 2. These profiles were generally similar to those previously reported for pharmaceuticals in river water and wastewater samples [63,69,70].

### 2.5. Monitoring Antimicrobials in Hospital Wastewater

Wastewater was collected from a general hospital in an urban area of Japan with over 500 beds and that dealt with an average of more than 1500 patients per day. Hospital wastewater is discharged into the municipal sewage system, where it merges with domestic and industrial wastewater before being introduced into the WWTP. Sampling was conducted between January 2021 and May 2023 at a frequency of approximately once per year for approximately two and a half years. A total of 26 wastewater samples were collected: in 2021, 10 samples were collected from January, and April to December; in 2022, 11 samples were collected from February to December; and in 2023, five samples were collected from January to May. A stainless-steel ladle was used to collect hospital wastewater. The samples were then placed in sterile bottles. Composite samplers could not be installed to collect wastewater from hospitals. Therefore, manual sampling was performed at a fixed frequency [22,71]. All samples were immediately transported to the laboratory in a cooler box (within 2 h), frozen at –20 °C until analysis, and thawed at 4 °C in the dark for the experiment.

### 2.6. Statistical Analysis

The data for the tested traits were analysed using Microsoft Excel and presented as mean values and their individual standard deviations. A paired *t*-test was conducted to evaluate the differences in inactivation rates between water samples, with a statistical significance of *p* < 0.05.

## 3. Results and Discussion

### 3.1. Effect of Solid-Phase Sample Loading on the Analysis of Antimicrobials in Wastewater Using a 96-Well SPE Plate

**Figure 2:**
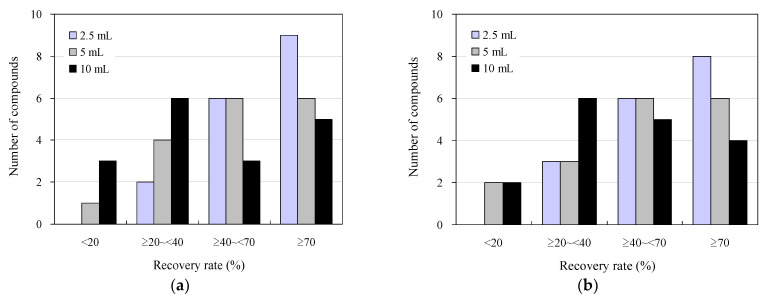
Effect of sample loading on the solid phase in addition to recovery studies under wastewater antimicrobial determination conditions using 96-well SPE plates: (**a**) 30 mg and (**b**) 60 mg solid-phase carrier.

Figure 2 displays the results of the addition-recovery tests using 96-well SPE plates with 30 mg and 60 mg solid-phase carriers, with varying sample loading volumes to the solid phase. Among the wastewater sample loading conditions examined, the highest recovery was observed when 2.5 mL was loaded, with nine compounds exhibiting recoveries of 70% or greater for both 30 and 60 mg solid-phase carriers. Additionally, five and four compounds showed recoveries of 40–70%, whereas one and two compounds showed recoveries of 20–40%, respectively. No compounds exhibited recoveries below 20%. As the volume of the wastewater samples loaded on the solid phase increased, the recovery rate decreased for both the 30 mg and 60 mg solid-phase carriers. Specifically, when 10 mL was loaded, four and two compounds with recoveries of ≥70% were observed for the 30 mg and 60 mg solid-phase carriers, respectively. Moreover, three and two compounds with recoveries of ≤20% were observed for the 30 mg and 60 mg solid-phase carriers, respectively. This indicates that the sample was not retained in the solid phase beyond its retention limit, leading to a breakthrough phenomenon that occurred when water passed through the solid phase. [72,73].

These results align closely with previous findings regarding the distribution of recoveries of antimicrobials and other pharmaceuticals in environmental waters, determined manually using syringe-barrel SPE cartridges (40–110%) [63,74,75], human serum using 96-well SPE plates (20–110%) [50], and organic contaminants in human serum using 96-well SPE plates (20–80%). The results were similar to those obtained for organic contaminants (20–110%) and psychotropics in environmental waters (30–80%) [43], using 96-well SPE plates. Similarly, the results were comparable in accuracy to those obtained by conventional methods and previous reports. In conventional manual measurements using syringe-barrel SPE cartridges (200 mg solid-phase carrier volume), the sample loading volume was approximately 10 mL, and the solution was concentrated 50-fold after SPE for LC–MS/MS analysis. Conversely, in the measurements using the 96-well SPE plate, the volume of the effluent sample loaded onto the solid phase was reduced to less than 2.5 mL. For 96-well SPE plates, it is conceivable that the recovery rate could be improved by reducing the volume of the effluent sample loaded onto the solid phase to 2.5 mL or less. However, considering the minimum volume required for LC–MS/MS analysis (500 µL for a typical commercially available LC–MS/MS vial and approximately 100 µL for a trace volume vial), a decrease in the loading volume of the effluent sample will result in a decrease in the concentration factor. This decrease could lead to an increase in the lower LOD and lower LOQ, potentially hindering the highly sensitive detection of antimicrobials present in environmental samples at trace concentrations of ng/L [76,77]. Therefore, to strike a balance between the development of a highly accurate measurement system that enables rapid mass analysis and practical convenience [48,78], we conducted the following studies with a 2.5 mL sample loading volume on the solid phase.

The effect of filtration on the additive recovery test in the previous SPE step was also investigated, and the results suggested the possibility of maintaining the accuracy of the measurement even when the effluent passed directly through the solid phase without filtration (Figure S1). These results may be advantageous for the experimental operation of the syringe-barrel-type SPE cartridge because the solid phase becomes clogged due to the large sample loading volume (tens of millilitres), preventing the sample water from passing through the solid phase and subsequent experimental operations [50,72,73]. Furthermore, in the measurement of antimicrobials in environmental waters, because the target substances are highly hydrophilic and almost all are distributed in the filtrate as they are pharmaceutical compounds, the measurement was performed on the filtrate after filtration [79,80]. However, for pharmaceutical compounds with high hydrophobicity, reports exist on the development of analytical methods and studies on the actual conditions of activated sludge in sewage treatment plants and bottom sediments in river. These studies aimed to separate and measure antimicrobials in dissolved and suspended forms, targeting the residue of suspended matter on filter paper [81,82]. Given the above considerations, the ability to simultaneously analyse both dissolved and suspended forms of antimicrobials can be advantageous in terms of expediting and simplifying experimental procedures and conducting more comprehensive monitoring of antimicrobials in environmental waters.

### 3.2. Effect of Organic Solvent Composition on Eluent Volume and UPLC–MS/MS Analysis in the Detection of Antimicrobials in Wastewater Using a 96-Well SPE Plate

**Figure 3:**
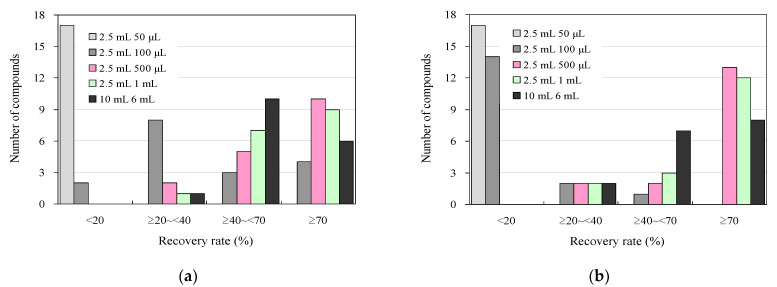
Influence of eluent volume in additive recovery studies under wastewater antimicrobial analysis conditions using 96-well SPE plates and comparison with syringe barrel SPE using conventional methods: (**a**) 30 mg and (**b**) 60 mg solid-phase carrier.

Figure 3 shows the results of the addition-recovery assays conducted using 96-well SPE plates with 30 mg and 60 mg solid-phase carriers, varying the volume of eluate that passed through the solid phase during the elution of target antimicrobials after SPE. For eluate volumes of 50 μL and 100 μL, the recoveries were 15 and 2 compounds for 30 mg of solid-phase carrier and 15 and 13 compounds for 60 mg of solid-phase carrier, respectively. This result indicates that most of the antimicrobials were not eluted and distributed in the solid phase and were not present in the eluate. However, when the eluate volume was increased to 500 µL and 1000 µL, the number of compounds with recoveries of 70% or greater tended to increase to 9–10 compounds at 30 mg solid-phase carrier and 10–12 compounds at 60 mg, with 4–6 compounds and 2–3 compounds showing recoveries between 40% and 70%. The recovery rate generally tended to increase as the number of extractions increased. Therefore, we also examined the effect of 500 μL of total eluate passed through the solid phase once versus passing 250 μL twice. However, we observed no improvement in recovery between the two cases (Figure S2). These findings suggest that efficient elution can be achieved in a single elution operation by passing a sufficient amount of eluent through the solid phase using a 96-well SPE plate. Incidentally, collection plates for collecting the eluate from the solid phase that is compatible with Extration+ and suitable for measuring trace samples by UPLC–MS/MS are currently commercially available in 700 μL or 800 μL volumes [60]. When the elution solution volume was 1000 μL, each solution was concentrated using two collection plates, as described above. However, consolidating these solutions into one is a complex procedure. Therefore, 500 μL of elution solution was used in the solid phase to simplify the experimental operation.

The effect of dissolving the solid-phase extracted solution in the final solvent after gentle concentration and solidification with nitrogen gas on the peak area of each antibacterial agent was investigated by changing the ratio of 0.1% aqueous formic acid solution to methanol, which was used for UPLC–MS/MS analysis. The peak area was largest when the ratio of 0.1% formic acid solution to methanol was 90:10, and the peak area decreased as the ratio of methanol increased (Figure S3). In the 25% methanol condition, one compound had a peak area that decreased to 70% or less; in the 40% methanol condition, three compounds had a peak area of 70% or less; in the 55% and 70% methanol conditions, six and eight compounds, respectively; in the 85% and 100% methanol conditions, 11 and 13 compounds had a peak area of 20% or less, respectively; and five compounds had a peak area of 20% or less. The decrease in the peak area with an increasing percentage of organic solvent in the final solvent may be due to the high polarity of the target antimicrobials, which reduces the UPLC column distribution and MS/MS ionisation efficiency when measured using the reversed-layer column used in this study. The polarities of the two antimicrobials can be found in [30,77,83]. Based on these results, it is necessary to replace the final solvent with a high proportion of aqueous solvent after elution with 100% methanol and acetone in the SPE process, with a final solvent that has a high proportion of aqueous solvent after concentrating and drying the solution with nitrogen gas to perform accurate measurements. Multiple operations were performed during concentration and solidification using nitrogen gas. In the process of concentration and solidification using nitrogen gas, multiple operations or concentration and solidification using a concentration and solidification unit corresponding to a 96-well plate [84,85] are considered necessary. With recent developments in science and technology and the progress of HTA research and development, the development of convenient instruments and the spread of instruments that directly inject LC–MS/MS online have progressed, and further accumulation of knowledge and integration following technological improvements will be necessary in the future. The integration of these systems is necessary in the future because more knowledge is accumulated and technologies are improved [86,87].

### 3.3. Application to Monitoring of Antimicrobials in Hospital Wastewater

**Figure 4:**
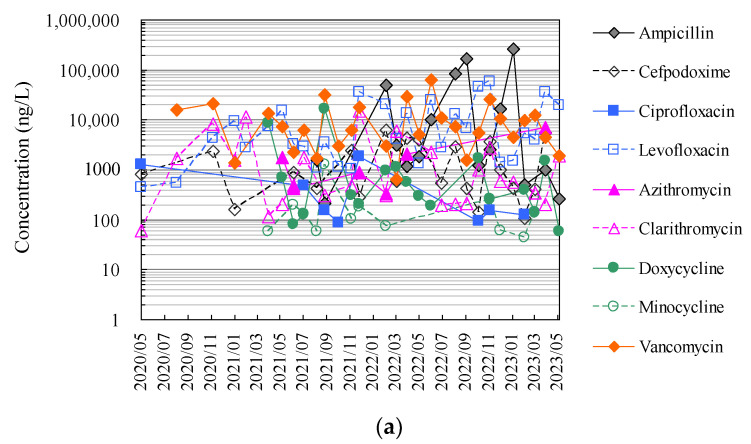

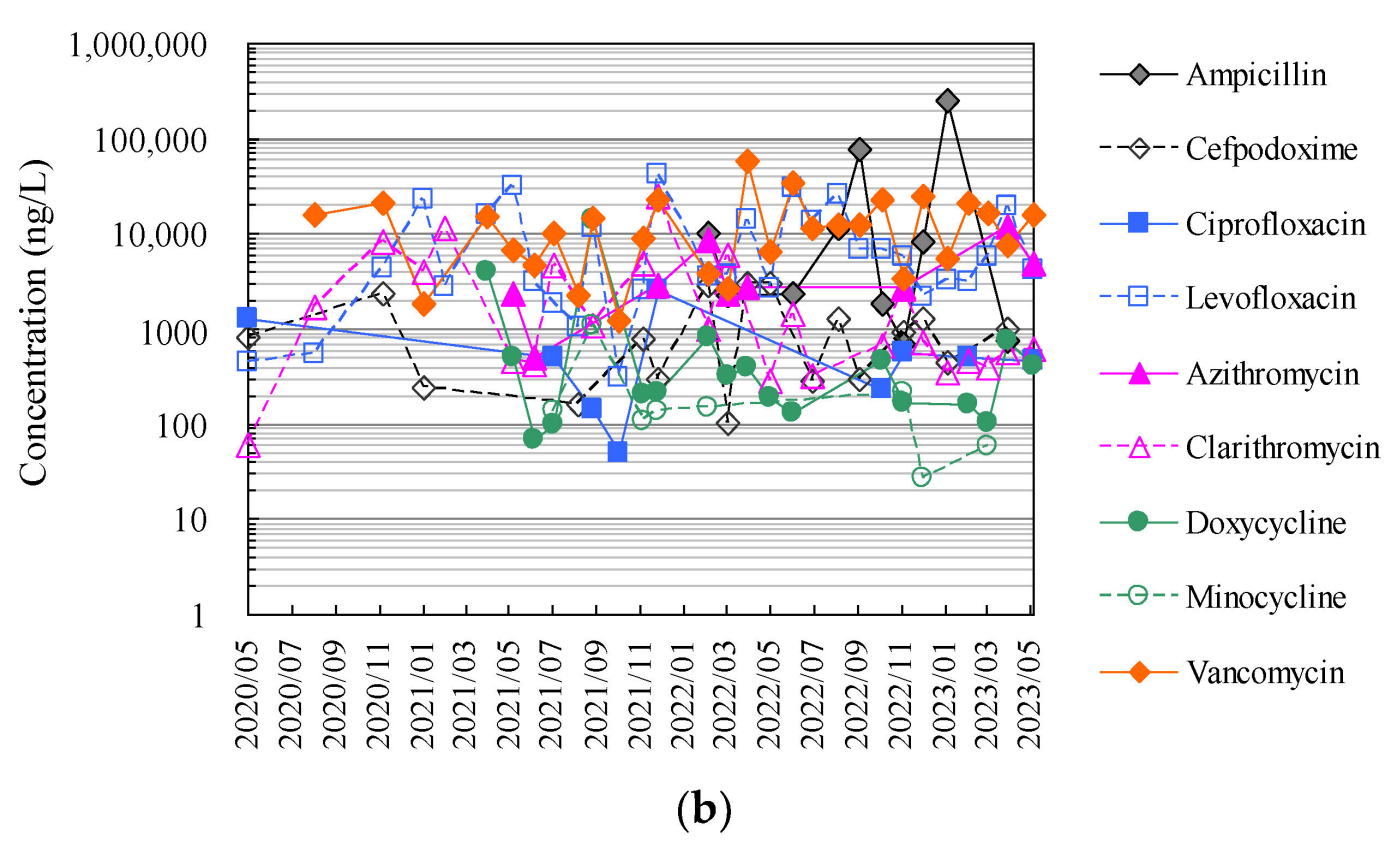
Evaluation of correspondence between manual analysis of hospital wastewater samples by conventional method and HTA developed in this study: (**a**) results of manual analysis by conventional method and (**b**) results of assay by HTA in this study.

Finally, Figure 4 and Tables S1 and S2 show the results of the analysis of antimicrobials in hospital wastewater over approximately 2.5 years using the HTA developed in this study, which makes full use of an automated pipetting system and an automated SPE pre-treatment system, as well as the results of the manual analysis using conventional methods.

The HTA detected nine antimicrobials (ampicillin, cefpodoxime, ciprofloxacin, levofloxacin, azithromycin, clarithromycin, doxycycline, minocycline, and vancomycin) in hospital wastewater at concentrations ranging from 27 ng/L (minocycline) to 24.8 μg/L (ampicillin). The detection frequencies in hospital wastewater ranged from 31 to 100% for ampicillin, ciprofloxacin, azithromycin, and minocycline; 58–77% for cefpodoxime, clarithromycin, and doxycycline; and 58–77% for levofloxacin and doxycycline. Levofloxacin and vancomycin were detected in all effluent samples throughout the sampling period [52]. The antimicrobials detected in hospital wastewater were likely derived from their use in the hospitals of interest. These concentrations were generally one order of magnitude, similar to previous reports investigating antimicrobials in hospital wastewater from urban areas in Japan [69,75]. Among these antimicrobials detected in hospital wastewater, ampicillin, levofloxacin, and vancomycin tended to be detected at mean concentrations of 36.3 μg/L ± 78.0 μg/L, 10.9 μg/L ± 11.3 μg/L, and 13.4 μg/L ± 12.3 μg/L, respectively, and tended to be detected at concentrations approximately one to two orders of magnitude higher than those of other antimicrobials. Benzylpenicillin, cefdinir, cefpodoxime proxetil, ceftiofur, enrofloxacin, chlortetracycline, oxytetracycline, and tetracycline were not detected in the effluent. Previous reports have reported that some antimicrobials are not detected in wastewater, which would be related to the fact that these antimicrobials were not in use at the hospital at the time of the wastewater sampling in the survey [88,89]. *β*-lactam antimicrobials are readily hydrolysed and disappear from wastewater within a few hours [90,91].

**Figure 5:**
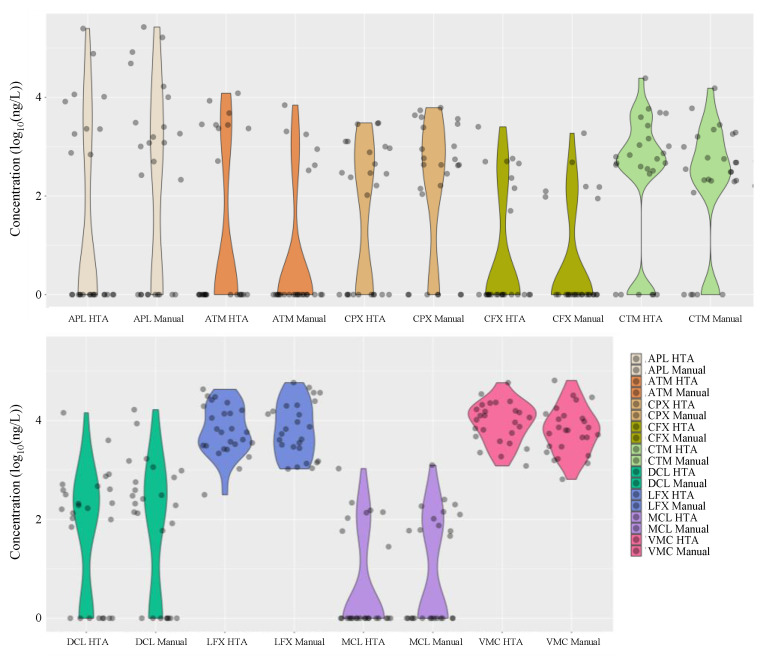
Distribution of mean concentrations of various antimicrobials detected in hospital wastewaters after manual analysis using conventional methods and adaptation of the HTA developed in this study (*n* = 26).

Figure 5 summarises the distribution of the detected antimicrobial concentrations in hospital wastewater during the entire study period. Interestingly, the results of manual analysis using conventional methods were similar to those obtained with HTA, with nine antimicrobials (ampicillin, cefpodoxime, ciprofloxacin, levofloxacin, azithromycin, clarithromycin, doxycycline, minocycline, and vancomycin) detected in the hospital wastewater at concentrations ranging from 45 ng/L to 26.7 μg/L. Azithromycin, clarithromycin, doxycycline, minocycline, and vancomycin were detected in hospital wastewater at concentrations ranging from 45 ng/L to 26.7 μg/L. The detection frequencies in the hospital wastewater ranged from 27% to 100%, with similar results for each compound.

**Figure 6:**
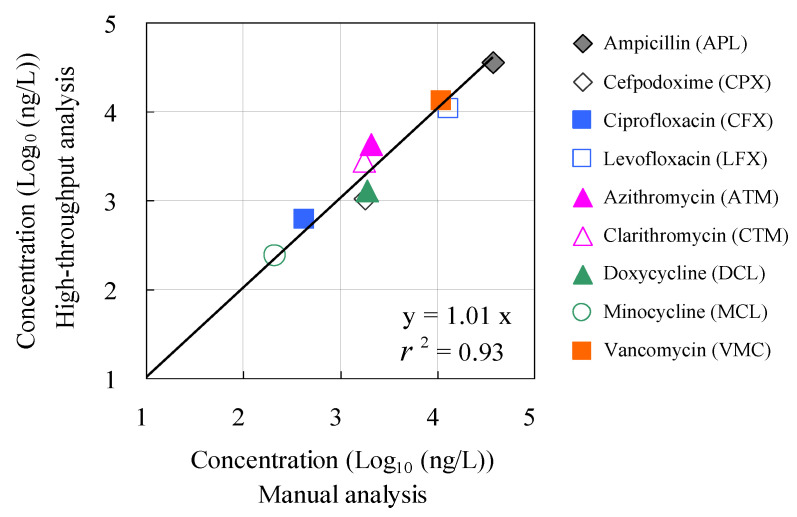
Relationship between corresponding concentration values determined by manual analysis and concentration values determined by the HTA developed in this study.

Figure 6 shows the results of the agreement evaluation between the concentrations of antimicrobials detected by manual analysis using conventional methods and those detected by the HTA system. No statistically significant differences were observed (*p* > 0.05) between the measured concentrations based on the conventional manual method and those based on the HTA, except for ampicillin and cefpodoxime. The agreement ratios between the measured concentrations based on the conventional manual method and HTA were plotted against each other. A positive correlation was observed between these two parameters (*r* = 0.99). These results suggest the effectiveness of HTA that combines an automated pipetting system and an automated SPE system for rapid, high-volume analysis of antimicrobials in environmental waters. To our knowledge, this is the first report to evaluate the accuracy and agreement between concentrations based on manual analysis and those measured with HTA in hospital wastewater. These findings provide a comprehensive understanding of the environmental risks associated with antimicrobials in aquatic environments.

The system for measuring antimicrobials in wastewater using a 96-well SPE plate with automated pipetting and an SPE system has the advantage of being more rapid than a syringe-barrel-type SPE cartridge, which is mainly used in conventional manual analysis, as each sample is measured individually. Additionally, the accuracy of the assay is equivalent to or slightly better than that of manual analysis and is an effective means of reducing human error; moreover it is more efficient, saving labour in HTA [39,92]. In addition, the 96-well SPE plate allows the use of a single plate instead of the large number of SPE cartridges and glass vials required for each sample in manual LC–MS/MS measurements, conserving resources and reduces waste and contributing to the cost of ownership of the measurements. In fact, the cost of consumables such as organic solvents, SPE, and glass vials required for the sample pre-treatment supplies used in the present investigation was approximately US $940 for manual analysis, compared to $600 for the HTA system, a cost reduction of more than 30%. Furthermore, the time required for pre-treatment of environmental water samples is approximately 6 to 7 h in the case of manual analysis and 3 to 4 h in the case of the HTA system, which can also be expected to reduce workload and improve efficiency. This conserves resources and reduces waste, contributing to the cost of goods and human workload and efficiency. These results suggest that HTA is an effective approach for monitoring antimicrobials in environmental waters.

Previous studies have reported that antimicrobials found in hospital wastewater are also found in the influent water of WWTPs at levels ranging from tens of ng/L to several μg/L and in river water in the ng/L range [93,94]. Antimicrobials present in wastewater are partially removed during the water treatment process at WWTPs; however, complete removal tends to be difficult at existing WWTPs, and antimicrobials enter the river environment [54,95]. These findings support the possibility that more advanced wastewater treatment in WWTPs, as well as the implementation of advanced wastewater treatment systems in hospitals and other healthcare facilities, could be effective measures to reduce the risk of nosocomial infections and the environmental impacts associated with AMR [96,97].

Research on the appropriate treatment of hospital wastewater is progressing worldwide. Studies have attempted to evaluate and verify the effectiveness of advanced wastewater treatment systems in hospital facilities, starting with laboratory-scale investigations [2,53,98]. In addition to continuing to gather knowledge through further detailed studies, HTA, which makes full use of the combination of automated pipetting systems and automated SPE systems studied in this research project, will be adapted for monitoring considering regional characteristics, and its effectiveness will be verified. Baseline information that will lead to a nationwide monitoring survey should be gathered for detailed risk assessment and management of the environmental AMR impact of antimicrobials in aquatic environments.

## 4. Conclusions

In the present study, a rapid mass analysis method for measuring antimicrobials in environmental water was developed. For the development of a high-throughput analysis system for residual antimicrobials in wastewater in this study, we are using the latest automated pipetting and solid-phase extraction instruments that have recently become available on the market. However, these are instruments, and detailed measurement conditions and optimal analytical methods will be established through actual research. The present results in the development of a high-throughput analysis system that is expected to be highly convenient and effective in practice is a significant achievement for future monitoring, which is essential for understanding the actual status of antimicrobial resistance in the environment. The effectiveness of this method for nationwide monitoring is based on hospital wastewater monitoring. The present method for measuring antimicrobials in environmental water with high precision and efficiency will contribute to developing similar countermeasures in Japan and in developed countries, such as Europe and the United States, as well as in rapidly developing countries. Additionally, this method can contribute to AMR action plans and the One Health Approach mandated by the WHO.

Nationwide monitoring of AMR in environmental waters is useful for understanding the status of AMR in the environment and is expected to provide a comprehensive understanding of the various problems associated with AMRB in jurisdictional areas. The results of this study are expected to be useful in establishing a system for the nationwide development of environmental AMR monitoring surveys and conducting trend surveys to clarify the current status of the environmental burden of AMR. Our findings can help enhance the effectiveness of environmental AMR monitoring systems for antimicrobials in aquatic environments and the implementation of advanced wastewater treatment systems at both WWTPs and medical facilities, thereby contributing to environmental safety and human health.

## Figures and Tables

**Table 2:** LC–MS/MS parameters and validations of each antimicrobial.

Classification	Compound	Ionisation Mode	Precursor Ion (*m*/*z*)	Product Ion (*m*/*z*)	Cone Voltage (V)	Collision Energy (eV)	Manual Analysis			HTA		
							LOD (ng/L)	LOQ (ng/L)	Reproducibility (%)	LOD (ng/L)	LOQ (ng/L)	Reproducibility (%)
*β*-lactams	Ampicillin	ESI+	350.2	*105.9*, 192.0	29	24	1.6	5.4	8.7	3.2	10.7	6.6
	Benzylpenicillin	ESI+	335.2	*160.1*, 174.0	32	23	0.5	1.7	8.3	1.0	3.3	4.6
	Cefdinir	ESI+	369.2	170.0, *227.0*	30	20	0.2	0.8	8.6	0.5	1.6	5.7
	Cefpodoxime	ESI+	427.8	*240.5*, 395.9	31	15	0.3	1.1	6.1	0.6	2.1	13.1
	Cefpodoxime proxetil	ESI+	557.5	*409.8*, 525.2	30	18	1.2	3.9	6.9	2.4	7.9	5.7
	Ceftiofur	ESI+	524.1	*240.9*	38	18	0.6	2.1	4.4	1.3	4.3	1.6
New quinolones	Ciprofloxacin	ESI+	332.2	*288.2*, 314.2	40	25	0.7	2.2	5.0	1.3	4.5	6.0
	Enrofloxacin	ESI+	360.0	*316.2*, 245.2	37	19	0.2	0.8	7.8	0.5	1.5	5.8
	Levofloxacin	ESI+	362.2	261.2, *318.2*	40	21	0.3	1.0	11.5	0.6	2.0	5.2
Macrolides	Azithromycin	ESI+	350.2	*105.9*, 192.0	29	24	0.3	1.1	8.3	0.6	2.1	7.8
	Clarithromycin	ESI+	748.2	316.6, *558.3*	38	18	0.5	1.6	8.4	0.9	3.1	9.0
Tetracyclines	Chlortetracycline	ESI+	479.2	443.5, *461.5*	36	20	0.3	1.1	2.1	0.6	2.1	1.0
	Doxycycline	ESI+	445.2	*428.3*	32	18	0.3	1.0	3.1	0.6	2.0	3.8
	Minocycline	ESI+	458.3	*441.0*	36	21	0.3	1.0	2.2	0.6	2.0	10.3
	Oxytetracycline	ESI+	461.2	*425.8*	28	19	0.3	1.1	2.5	0.6	2.1	8.7
	Tetracycline	ESI+	445.2	*409.9*, 427.1	28	20	0.4	1.3	3.1	0.8	2.5	9.6
Glycopeptide	Vancomycin	ESI+	724.2	82.9, *100.2*	17	18	0.6	1.9	9.4	1.1	3.8	8.1

(product ions in italics are used for quantification).

## Data Availability

The data presented in this study are available on request from the corresponding author.

## Supplementary Materials

The following supporting information can be downloaded from https://www.mdpi.com/2079-6382/13/4/335/s1, Figure S1. Effects of filtration manipulation and recovery under conditions of antimicrobial determination in wastewater using 96-well SPE plates; Figure S2. Influence of elution frequency and recovery studies under wastewater antimicrobial determination conditions using 96-well SPE plates and comparison with syringe barrel SPE using conventional methods: (a) one-time extraction and (b) twice extraction; Figure S3. Effect of final solvent composition on the peak area of each antimicrobial in the LC–MS/MS measurements; Table S1. The presence of antimicrobials in the hospital wastewater was based on the manual analysis by manual analysis using a syringe-barrel SPE cartridge; Table S2. The occurrence of antimicrobials in hospital wastewater based on HTA using 96-well plate-type SPE was developed in this study using a combination of an automated pipetting system and an automated SPE pre-treatment system.
