# Supplementary material for: Development of a High-Throughput Analytical Method for Antimicrobials in Wastewater Using an Automated Pipetting and Solid-Phase Extraction System
Source: Antibiotics (Basel). 2024 Apr 5;13(4):335. doi: 10.3390/antibiotics13040335 (PMC11605239; doi:10.3390/antibiotics13040335)
Supplement: Supplementary file 1 [file antibiotics-13-00335-s001.zip › antibiotics-2924042-supplementary.pdf]

Supplemental Information for

Development of a High-throughput Analytical Method for Antimicrobials in Wastewater using an Automated Pipetting and Solid-Phase Extraction System

Author names: Takashi Azuma [1,*], Nobuaki Matsunaga [2], Norio Ohmagari [2,3], Makoto Kuroda [4,*]

Affiliation:

[1] Department of Pharmacy, Osaka Medical and Pharmaceutical University, Takatsuki 569-1094, Japan

[2] AMR Clinical Reference Center, National Center for Global Health and Medicine, Tokyo 162-8655, Japan

[3] Disease Control and Prevention Center, National Center for Global Health and Medicine, Tokyo 162-8655, Japan

[4] Pathogen Genomics Center, National Institute of Infectious Diseases, Tokyo 162-8640, Japan

*Corresponding author: Takashi Azuma

Affiliation: Department of Pharmacy, Osaka Medical and Pharmaceutical University, 4-20-1 Nasahara, Takatsuki, Osaka 569-1094, Japan

Tel: +81-72-690-1055, Fax: +81-72-690-1055

e-mail address: takashi.azuma@ompu.ac.jp, t.azuma.mail@gmail.com

*Corresponding author: Makoto Kuroda

Affiliation: Pathogen Genomics Center, National Institute of Infectious Diseases, Toyama 1-23-1, Shinjuku-ku, Tokyo 162-8640, Japan

Tell: +81-3-5285-1111, Fax: +81-3-5285-1150

e-mail address: makokuro@niid.go.jp

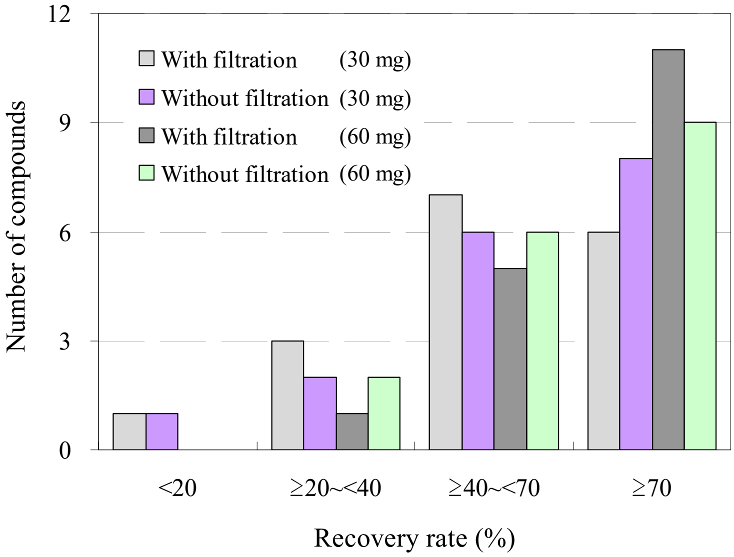

Figure S1. Effect of filtration manipulation and recovery under conditions of antimicrobial determination in wastewater using 96-well SPE plates.

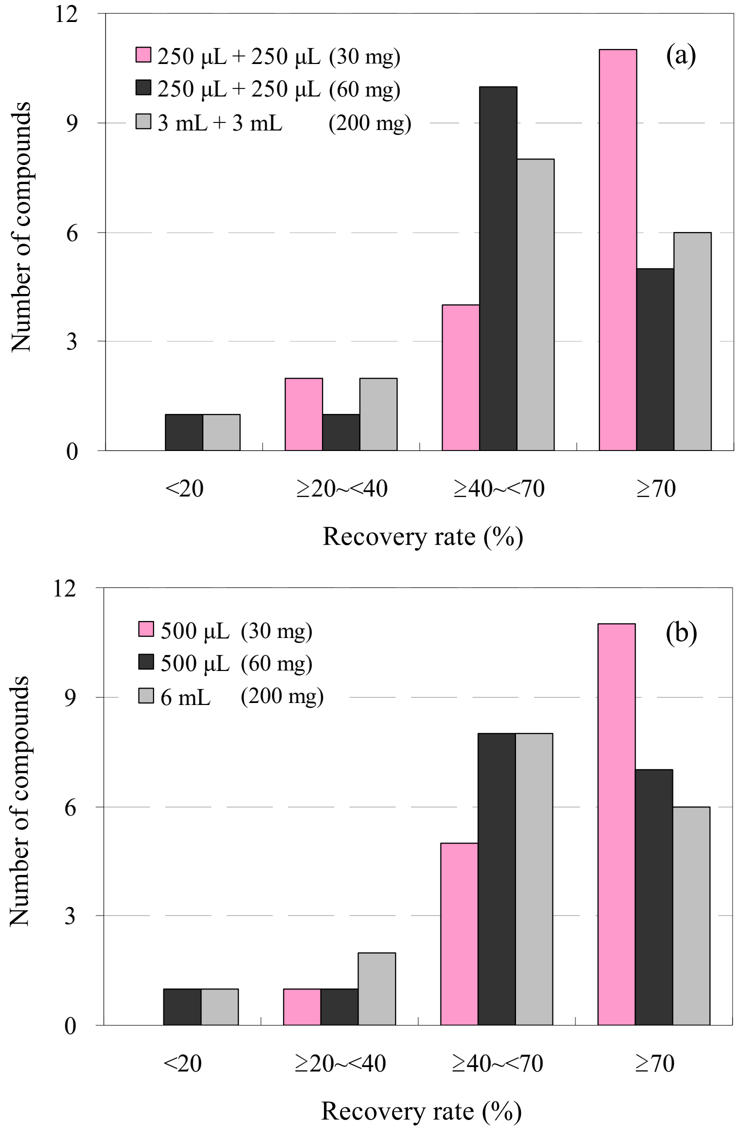

Figure S2. Influence of elution frequency and recovery studies under wastewater antimicrobial deter-mination conditions using 96-well SPE plates and comparison with syringe barrel SPE using conventional methods: (a) one-time extraction and (b) twice extraction.

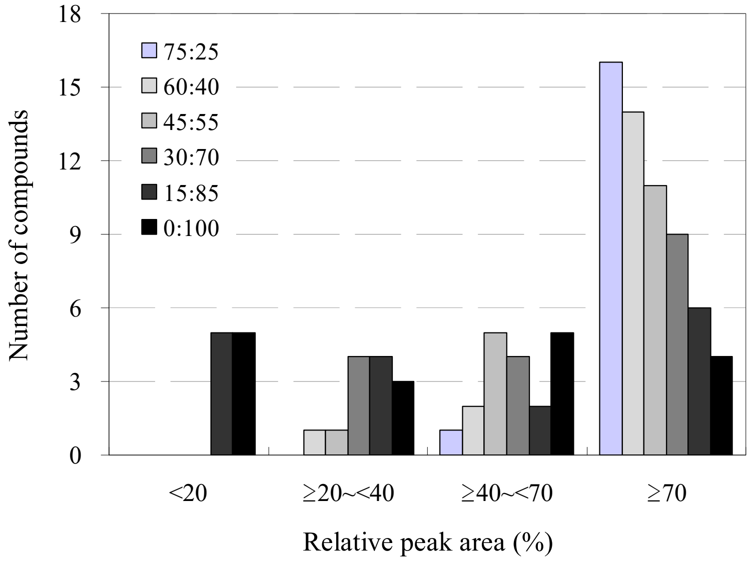

Figure S3. Effect of final solvent composition on the peak area of each antimicrobial in the LC-MS/MS measurements.

Table S1. The presence of antimicrobials in the hospital wastewater was based on the manual analysis by manual analysis using a syringe-barrel SPE cartridge.

Antimicrobials	2021										2022											2023					Mean (SD)	Max	Min	Frequency (%)
	0126	0426	0531	0629	0727	0830	0921	1026	1129	1221	0228	0328	0425	0525	0627	0725	0829	0928	1031	1129	1226	0130	0228	0327	0424	0529				
Ampicillin	N.D.	N.D.	N.D.	N.D.	N.D.	1,570	213	N.D.	N.D.	N.D.	48,694	3,069	1,191	1,836	10,063	N.D.	82,844	164,030	1,204	2,508	16,594	266,914	498	N.D.	1,008	263	37,656 (75,321)	266,914	213	100
Benzylpenicillin	N.D.	N.D.	N.D.	N.D.	N.D.	N.D.	N.D.	N.D.	N.D.	N.D.	N.D.	N.D.	N.D.	N.D.	N.D.	N.D.	N.D.	N.D.	N.D.	N.D.	N.D.	N.D.	N.D.	N.D.	N.D.	N.D.	N.A.	N.A.	N.A.	N.A.
Cefdinir	N.D.	N.D.	N.D.	N.D.	N.D.	N.D.	N.D.	N.D.	N.D.	N.D.	N.D.	N.D.	N.D.	N.D.	N.D.	N.D.	N.D.	N.D.	N.D.	N.D.	N.D.	N.D.	N.D.	N.D.	N.D.	N.D.	N.A.	N.A.	N.A.	N.A.
Cefpodoxime	161	N.D.	N.D.	890	N.D.	430	N.D.	N.D.	2,455	281	6,165	581	4,334	3,968	N.D.	553	2,883	427	140	3,662	1,018	426	108	415	5,458	N.D.	1,808 (1,990)	6,165	108	100
Cefpodoxime Proxetil	N.D.	N.D.	N.D.	N.D.	N.D.	N.D.	N.D.	N.D.	N.D.	N.D.	N.D.	N.D.	N.D.	N.D.	N.D.	N.D.	N.D.	N.D.	N.D.	N.D.	N.D.	N.D.	N.D.	N.D.	N.D.	N.D.	N.A.	N.A.	N.A.	N.A.
Ceftiofur	N.D.	N.D.	N.D.	N.D.	N.D.	N.D.	N.D.	N.D.	N.D.	N.D.	N.D.	N.D.	N.D.	N.D.	N.D.	N.D.	N.D.	N.D.	N.D.	N.D.	N.D.	N.D.	N.D.	N.D.	N.D.	N.D.	N.A.	N.A.	N.A.	N.A.
Ciprofloxacin	N.D.	N.D.	N.D.	N.D.	481	N.D.	154	88	N.D.	1,872	N.D.	N.D.	N.D.	N.D.	N.D.	N.D.	N.D.	N.D.	95	151	N.D.	N.D.	124	N.D.	N.D.	N.D.	423 (653)	1,872	88	100
Enrofloxacin	N.D.	N.D.	N.D.	N.D.	N.D.	N.D.	N.D.	N.D.	N.D.	N.D.	N.D.	N.D.	N.D.	N.D.	N.D.	N.D.	N.D.	N.D.	N.D.	N.D.	N.D.	N.D.	N.D.	N.D.	N.D.	N.D.	N.A.	N.A.	N.A.	N.A.
Levofloxacin	9,337	7,441	15,308	3,209	2,926	1,080	3,525	1,137	1,045	36,448	20,354	4,162	13,504	1,346	24,524	2,749	13,054	6,696	46,320	58,247	1,408	1,503	5,350	4,058	36,380	19,739	13,110 (15,508)	58,247	1,045	100
Azithromycin	N.D.	N.D.	1,774	420	N.D.	N.D.	N.D.	N.D.	N.D.	891	329	N.D.	2,037	N.D.	N.D.	N.D.	N.D.	N.D.	N.D.	N.D.	N.D.	N.D.	N.D.	N.D.	6,948	N.D.	2,066 (2,491)	6,948	329	100
Clarithromycin	1,590	116	205	476	1,803	N.D.	305	N.D.	475	15,310	307	6,003	N.D.	N.D.	2,215	195	202	216	990	2,768	595	564	N.D.	349	209	1,930	1,754 (3,395)	15,310	116	100
Chlortetracycline	N.D.	N.D.	N.D.	N.D.	N.D.	N.D.	N.D.	N.D.	N.D.	N.D.	N.D.	N.D.	N.D.	N.D.	N.D.	N.D.	N.D.	N.D.	N.D.	N.D.	N.D.	N.D.	N.D.	N.D.	N.D.	N.D.	N.A.	N.A.	N.A.	N.A.
Doxycycline	N.D.	8,672	703	82	132	N.D.	16,519	N.D.	307	207	964	1,136	566	299	191	N.D.	N.D.	N.D.	1,676	258	N.D.	N.D.	389	139	1,516	58	1,878 (4,153)	16,519	58	100
Minocycline	N.D.	58	N.D.	198	124	57	1,251	N.D.	102	184	74	N.D.	N.D.	N.D.	N.D.	N.D.	N.D.	N.D.	N.D.	251	60	N.D.	45	141	N.D.	N.D.	212 (334)	1,251	45	100
Oxytetracycline	N.D.	N.D.	N.D.	N.D.	N.D.	N.D.	N.D.	N.D.	N.D.	N.D.	N.D.	N.D.	N.D.	N.D.	N.D.	N.D.	N.D.	N.D.	N.D.	N.D.	N.D.	N.D.	N.D.	N.D.	N.D.	N.D.	N.A.	N.A.	N.A.	N.A.
Tetracycline	N.D.	N.D.	N.D.	N.D.	N.D.	N.D.	N.D.	N.D.	N.D.	N.D.	N.D.	N.D.	N.D.	N.D.	N.D.	N.D.	N.D.	N.D.	N.D.	N.D.	N.D.	N.D.	N.D.	N.D.	N.D.	N.D.	N.A.	N.A.	N.A.	N.A.
Vancomycin	1,359	13,562	7,198	2,273	6,342	1,672	32,102	2,942	6,326	17,717	3,006	645	29,377	5,148	64,388	10,940	7,244	1,560	5,430	26,138	10,599	4,554	9,564	12,477	4,537	1,935	11,117 (13,860)	64,388	645	100

N.D.: Not detected, N.A.: Not available.

Table S2. The Occurrence of antimicrobials in hospital wastewater based on HTA using 96-well plate-type SPE was developed in this study, using a combination of an automated pipetting system and an automated SPE pre-treatment system.

Antimicrobials	2021										2022											2023					Mean (SD)	Max	Min	Frequency (%)
	0126	0426	0531	0629	0727	0830	0921	1026	1129	1221	0228	0328	0425	0525	0627	0725	0829	0928	1031	1129	1226	0130	0228	0327	0424	0529				
Ampicillin	N.D.	N.D.	N.D.	N.D.	N.D.	N.D.	N.D.	N.D.	N.D.	N.D.	10,281	2,276	N.D.	N.D.	2,303	N.D.	11,456	76,782	1,810	692	8,218	248,463	N.D.	N.D.	747	N.D.	36,303 (78,010)	248,463	692	100
Benzylpenicillin	N.D.	N.D.	N.D.	N.D.	N.D.	N.D.	N.D.	N.D.	N.D.	N.D.	N.D.	N.D.	N.D.	N.D.	N.D.	N.D.	N.D.	N.D.	N.D.	N.D.	N.D.	N.D.	N.D.	N.D.	N.D.	N.D.	N.A.	N.A.	N.A.	N.A.
Cefdinir	N.D.	N.D.	N.D.	N.D.	N.D.	N.D.	N.D.	N.D.	N.D.	N.D.	N.D.	N.D.	N.D.	N.D.	N.D.	N.D.	N.D.	N.D.	N.D.	N.D.	N.D.	N.D.	N.D.	N.D.	N.D.	N.D.	N.A.	N.A.	N.A.	N.A.
Cefpodoxime	239	N.D.	N.D.	N.D.	N.D.	162	N.D.	N.D.	769	291	2,863	103	2,957	3,029	N.D.	281	1,276	295	N.D.	929	1,276	445	N.D.	N.D.	1,002	N.D.	1,061 (1,051)	3,029	103	100
Cefpodoxime Proxetil	N.D.	N.D.	N.D.	N.D.	N.D.	N.D.	N.D.	N.D.	N.D.	N.D.	N.D.	N.D.	N.D.	N.D.	N.D.	N.D.	N.D.	N.D.	N.D.	N.D.	N.D.	N.D.	N.D.	N.D.	N.D.	N.D.	N.A.	N.A.	N.A.	N.A.
Ceftiofur	N.D.	N.D.	N.D.	N.D.	N.D.	N.D.	N.D.	N.D.	N.D.	N.D.	N.D.	N.D.	N.D.	N.D.	N.D.	N.D.	N.D.	N.D.	N.D.	N.D.	N.D.	N.D.	N.D.	N.D.	N.D.	N.D.	N.A.	N.A.	N.A.	N.A.
Ciprofloxacin	N.D.	N.D.	N.D.	N.D.	502	N.D.	143	49	N.D.	2,530	N.D.	N.D.	N.D.	N.D.	N.D.	N.D.	N.D.	N.D.	231	572	N.D.	N.D.	497	N.D.	N.D.	455	622 (794)	2,530	49	100
Enrofloxacin	N.D.	N.D.	N.D.	N.D.	N.D.	N.D.	N.D.	N.D.	N.D.	N.D.	N.D.	N.D.	N.D.	N.D.	N.D.	N.D.	N.D.	N.D.	N.D.	N.D.	N.D.	N.D.	N.D.	N.D.	N.D.	N.D.	N.A.	N.A.	N.A.	N.A.
Levofloxacin	22,948	16,104	31,142	3,126	1,827	1,048	11,235	313	2,582	42,747	3,524	3,718	13,867	2,643	29,962	13,705	26,004	6,729	6,807	5,755	2,157	3,304	3,073	5,783	19,402	4,088	10,907 (11,315)	42,747	313	100
Azithromycin	N.D.	N.D.	2,339	509	N.D.	N.D.	N.D.	N.D.	N.D.	2,828	8,507	2,346	2,755	N.D.	N.D.	N.D.	N.D.	N.D.	N.D.	2,763	N.D.	N.D.	N.D.	N.D.	12,095	4,786	4,325 (3,668)	12,095	509	100
Clarithromycin	3,962	N.D.	462	426	4,739	N.D.	1,078	N.D.	4,892	24,441	1,020	5,863	N.D.	280	1,459	323	N.D.	N.D.	675	2,669	734	353	465	394	565	622	2,771 (5,402)	24,441	280	100
Chlortetracycline	N.D.	N.D.	N.D.	N.D.	N.D.	N.D.	N.D.	N.D.	N.D.	N.D.	N.D.	N.D.	N.D.	N.D.	N.D.	N.D.	N.D.	N.D.	N.D.	N.D.	N.D.	N.D.	N.D.	N.D.	N.D.	N.D.	N.A.	N.A.	N.A.	N.A.
Doxycycline	N.D.	3,964	508	69	98	N.D.	14,273	N.D.	207	212	817	316	389	191	133	N.D.	N.D.	N.D.	468	167	N.D.	N.D.	159	104	742	405	1,290 (3,359)	14,273	69	100
Minocycline	N.D.	N.D.	N.D.	N.D.	139	N.D.	1,064	N.D.	105	135	151	N.D.	N.D.	N.D.	N.D.	N.D.	N.D.	N.D.	N.D.	216	27	N.D.	N.D.	57	N.D.	N.D.	237 (339)	1,064	27	100
Oxytetracycline	N.D.	N.D.	N.D.	N.D.	N.D.	N.D.	N.D.	N.D.	N.D.	N.D.	N.D.	N.D.	N.D.	N.D.	N.D.	N.D.	N.D.	N.D.	N.D.	N.D.	N.D.	N.D.	N.D.	N.D.	N.D.	N.D.	N.A.	N.A.	N.A.	N.A.
Tetracycline	N.D.	N.D.	N.D.	N.D.	N.D.	N.D.	N.D.	N.D.	N.D.	N.D.	N.D.	N.D.	N.D.	N.D.	N.D.	N.D.	N.D.	N.D.	N.D.	N.D.	N.D.	N.D.	N.D.	N.D.	N.D.	N.D.	N.A.	N.A.	N.A.	N.A.
Vancomycin	1,861	15,206	6,888	4,729	10,335	2,236	14,483	1,204	9,146	23,102	3,784	2,642	57,947	6,469	34,181	11,519	12,493	12,579	22,491	3,420	24,283	5,586	20,751	16,766	7,486	15,660	13,356 (12,321)	57,947	1,204	100

N.D.: Not detected, N.A.: Not available.
